# Inverse Correlation between Promoter Strength and Excision Activity in Class 1 Integrons

**DOI:** 10.1371/journal.pgen.1000793

**Published:** 2010-01-08

**Authors:** Thomas Jové, Sandra Da Re, François Denis, Didier Mazel, Marie-Cécile Ploy

**Affiliations:** 1 Université de Limoges, Faculté de Médecine, EA3175, Limoges, France; 2 INSERM, Equipe Avenir, Limoges, France; 3 Institut Pasteur, Unité Plasticité du Génome Bactérien, CNRS URA 2171, Paris, France; Universidad de Sevilla, Spain

## Abstract

Class 1 integrons are widespread genetic elements that allow bacteria to capture and express gene cassettes that are usually promoterless. These integrons play a major role in the dissemination of antibiotic resistance among Gram-negative bacteria. They typically consist of a gene (*intI*) encoding an integrase (that catalyzes the gene cassette movement by site-specific recombination), a recombination site (*attI1*), and a promoter (Pc) responsible for the expression of inserted gene cassettes. The Pc promoter can occasionally be combined with a second promoter designated P2, and several Pc variants with different strengths have been described, although their relative distribution is not known. The Pc promoter in class 1 integrons is located within the *intI1* coding sequence. The Pc polymorphism affects the amino acid sequence of IntI1 and the effect of this feature on the integrase recombination activity has not previously been investigated. We therefore conducted an extensive *in silico* study of class 1 integron sequences in order to assess the distribution of Pc variants. We also measured these promoters' strength by means of transcriptional reporter gene fusion experiments and estimated the excision and integration activities of the different IntI1 variants. We found that there are currently 13 Pc variants, leading to 10 IntI1 variants, that have a highly uneven distribution. There are five main Pc-P2 combinations, corresponding to five promoter strengths, and three main integrases displaying similar integration activity but very different excision efficiency. Promoter strength correlates with integrase excision activity: the weaker the promoter, the stronger the integrase. The tight relationship between the aptitude of class 1 integrons to recombine cassettes and express gene cassettes may be a key to understanding the short-term evolution of integrons. Dissemination of integron-driven drug resistance is therefore more complex than previously thought.

## Author Summary

Integrons are widespread bacterial genetic elements able to capture and express gene cassettes that often encode antibiotic resistance determinants. Gene cassettes are usually promoterless and are transcribed from a common promoter, Pc. Pc is located within the coding sequence of the integron integrase, IntI, which is the key element catalyzing the integration and excision of gene cassettes. Several Pc variants, associated with different integrase amino acid sequences, have been described, but the influence of these differences on integrase activity has never been investigated. Here, we show that Pc is highly polymorphic, conferring a wide range of antibiotic resistance. Furthermore, we found that different Pc variants are associated with different integrase excision activities: the weaker the Pc variant, the more active the integrase. These results point to evolutionary compromises between the expression and mobility of drug resistance determinants located on integrons.

## Introduction

**Figure 1:**
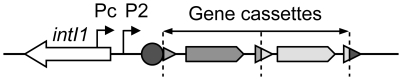
General structure of a class 1 integron. Coding sequences are indicated by arrows, *attC* cassette recombination sites by triangles, the integron recombination site *attI1* by a circle, and the gene cassette promoters Pc and P2 by broken arrows. Dotted vertical bars represent gene cassette boundaries.

Integrons are natural genetic elements that can acquire, exchange and express genes within gene cassettes. The integron platform is composed of a gene, *intI*, that encodes a site-specific recombinase, IntI, a recombination site, *attI*, and a functional promoter, Pc, divergent to the integrase gene [1] (Figure 1). Gene cassettes are small mobile units composed of one coding sequence and a recombination site, *attC*. Integrons exchange gene cassettes through integrase-catalyzed site-specific recombination between *attI* and *attC* sites, resulting in the insertion of the gene cassette at the *attI* site, or between two *attC* sites, leading to the excision of the gene cassette(s) from the gene cassette array [2]–[6]. Multi-resistant integrons (MRI) contain up to eight gene cassettes encoding antibiotic resistance. To date, more than 130 gene cassettes have been described, conferring resistance to almost all antibiotic classes [7]. MRI play a major role in the dissemination of antibiotic resistance among Gram-negative bacteria, through horizontal gene transfer [8]. Five classes of MRI have been described on the basis of the integrase coding sequence, class 1 being the most prevalent [8].

Gene cassettes are usually promoterless, and their genes are transcribed from the Pc promoter, as in an operon (Figure 1), the level of transcription depending on their position within the integron [9],[10]. Among class 1 MRIs, several Pc variants have been defined on the basis of their −35 and −10 hexamer sequences. Four Pc variants have been named according to their sequence homology with the σ^70^ promoter consensus and their estimated respective strengths, as follows: PcS for ‘Strong’, PcW for ‘Weak’ (PcS being 30-fold stronger than PcW), PcH1 for Hybrid 1 and PcH2 for Hybrid 2, these two latter Pc variants containing the −35 and −10 hexamers of PcW and PcS in opposite combinations (Table 1), and having intermediate strengths [11]–[13]. More recently, a new variant was reported to be significantly stronger than PcS [14], and we therefore named it ‘Super-Strong’ or PcSS. Three other Pc variants have been described but their strength has not been determined; for simplicity, we named these Pc promoters PcIn42, PcIn116 and PcPUO, as they are carried by integrons In42 and In116 and by plasmid pUO901, respectively [15]–[17]. Nesvera and co-workers found a C to G mutation 2 bp upstream of the −10 hexamer in PcW and showed that this mutation increased promoter efficiency by a factor of 5 [18]. This mutation creates a ‘TGN’ extended −10 motif that is known to increase the transcription efficiency of σ^70^ promoters in *E. coli* [19]. Also, class 1 integrons occasionally harbor a second functional promoter named P2, located in the *attI* site and created by the insertion of three G residues, optimizing the spacing (17 bp) between potential −35 and −10 hexamer sequences [9] (Figure 1). Given the diversity of Pc variants and the range of their respective strengths, an identical array of gene cassettes should be differently expressed depending on the Pc variant present in the integron platform. However, the distribution of Pc variants among the numerous class 1 integrons has never been comprehensively studied.

**Table 1:** Pc variants and P2 promoter sequences found in class 1 integrons.

Promoter	Variants	−35 sequence^a^	Spacer length (bp)	−10 sequence^a^^,^ b	Accession number or reference	Occurrence (%)
**Pc**	PcS (Strong)	TTGACA	17	TAAACT	U12441	24.3
	PcW (Weak)	T**G**GACA	17	TAA**G**CT	U49101	41.7
	PcH1 (Hybrid 1)	T**G**GACA	17	TAAACT	M95287	28
	PcH2 (Hybrid 2)	TTGACA	17	TAA**G**CT	U13880	4.4
	PcSS (Super-Strong)	TTGA**T**A	17	TAAACT	[14]	0.3
	PcIn42	TTG**G**CA	17	TAAACT	AJ243491	0.3
	PcIn116	TTGACA	17	T**G**AACT	AJ621187	0.3
	PcPUO	T**C**GACA	17	TAAACT	S68049	0.6
**P2**	P2	TTGTTA	17	TACAGT	U42226	8.7
	P2m1 (Mutated 1)	TTGTTA	17	gACAGT	DQ315788	0.6
	P2m2 (Mutated 2)	TTGTTA	17	TACAca	[30]	0.3

**a** Bases that differ from those of PcS are shown in bold.

**b** The mutated base in the P2 −10 hexamer sequence is indicated in lower case.

In class 1 MRIs, the Pc promoter is located within the integrase coding sequence (Figure 1). Some of the base substitutions in the −35 and/or −10 hexamer sequences defining the different Pc variants actually correlate with amino acid changes in the IntI1 sequence. These variations in the IntI1 protein sequence could potentially influence integrase recombination activity and define different IntI1 catalytic variants.

We first performed an extensive *in silico* examination of all class 1 integron sequences available in databases in order to determine the prevalence of Pc variants and, therefore, the prevalence of IntI1 variants. We then estimated the strength of all Pc variants and Pc-P2 combinations in the same reporter gene assay, as well as the excision and integration activity of the main IntI1 variants. We found a very unequal distribution of the Pc variants, and a negative correlation between the strength of the Pc variant and the recombination efficiency of the corresponding IntI1 protein.

## Results

### Distribution of the different gene cassette promoter variants

We analyzed the sequences of 321 distinct class 1 integrons containing the complete sequences of both gene cassette arrays and Pc-P2 promoters (see Materials and Methods). When considering only the −35 and −10 hexamer sequences, we found no more than the eight variants identified previously. However, their distribution was highly uneven, four variants (PcW, PcS, PcH1 and PcH2) totalling 98.4% of the sequences analyzed (Table 1). The most frequent Pc variant was PcW (41.7%), followed by PcH1 (28%), PcS (24.3%) and PcH2 (4.4%). The four other Pc variants, all more recently described, were extremely rare (Table 1). The most prevalent Pc variant among class 1 integrons appeared to be the weak PcW, but in 58% of the analyzed PcW-containing integrons this promoter was associated with either a ‘TGN’ extended −10 motif [20] (hereafter designated variant PcW_TGN-10_) or the second gene cassette promoter P2 (Table 2). These two features were much less frequent with the other Pc variants (Table 2). The dataset also contained two other extremely rare Pc configurations, designated PcW_TAN-10_ and PcH1_TTN-10_, in which the second base upstream of the −10 hexamer was replaced by an A or a T instead of C, respectively, as well as two other rare forms of P2, designated P2m1 and P2m2, for ‘P2 mutated form 1’ and ‘P2 mutated form 2’ (Table 1 and Table 2).

**Table 2:** Combinations of class 1 integron Pc-Pc2 sequences.

Pc variant	TXN-10 motif^a^	P2 form	Total number	Frequency (%)^b^	Occurrence (%)^c^
**PcS**	-	-	76	97.4	23.7
	TGN-10*	-	1	1.3	0.3
	-	P2	1	1.3	0.3
			**78**	**100**	**24.3**
**PcW**	-	-	53	39.6	16.5
	TGN-10*	-	54	40.3	16.8
	TGN-10*	P2	1	0.7	0.3
	TGN-10*	P2m1	1	0.7	0.3
	TAN-10	-	1	0.7	0.3
	-	P2	23	17.2	7.2
	-	P2m2	1	0.7	0.3
			**134**	**100**	**41.7**
**PcH1**	-	-	84	93.3	26.2
	TTN-10	-	2	2.2	0.6
	-	P2	3	3.3	0.9
		P2m1	1	1.1	0.3
	-		**90**	**100**	**28.0**
**PcH2**	-	-	9	64.3	2.8
	TGN-10*	-	5	35.7	1.6
			**14**	**100**	**4.4**
**PcPUO**	-	-	**2**	**100**	**0.6**
**PcIn42**	-	-	**1**	**100**	**0.3**
**PcIn116**	-	-	**1**	**100**	**0.3**
**PcSS**	-	-	**1**	**100**	**0.3**

**a** The TGN-10 configuration refers to an extended −10 promoter and is indicated by a star [19].

**b** Frequency of each Pc-P2 combination within the Pc variant group.

**c** Occurrence of each Pc-P2 combination among the 321 analyzed integrons.

Altogether, on the basis of the −35 and −10 hexamers and the sequence upstream of the −10 box, we identified 13 Pc variants, four of which were also found associated with a form of the P2 promoter (Table 2).

### Relative strengths of gene cassette promoter variants

**Figure 2:**
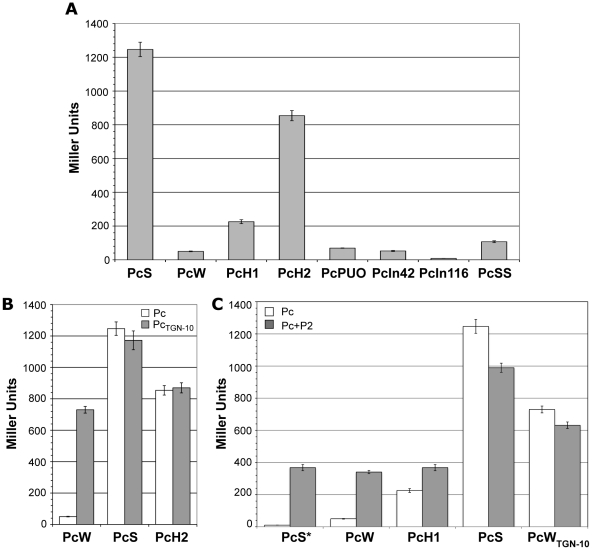
Strength of class 1 Pc variants. (A) Pc promoter strength was estimated by measuring the β-galactosidase activity of Pc-*lacZ* transcriptional fusions. (B) β-galactosidase activities of Pc-*lacZ* fusions for different Pc variants in either their wild-type configuration (white bars) or bearing the TGN-10 motif (grey bars). (C) Same as (B), but for Pc variants in combination with P2. PcS* designates an artificially mutated PcS variant that was combined with P2 to serve as a control for specific P2 promoter activity. At least five independent assays were performed for each variant and in each experiment. Error bars indicate the standard error of the mean.

Until recently, the promoter strength of only 4 of the 8 known variants (PcSS, PcS, PcH1 and PcW) had been estimated, but variants strength had never been compared in the same assay [11],[14]. We therefore examined the capacity of all the Pc variants and the different Pc-P2 configurations to drive the expression of the *lacZ* reporter gene cloned in a transcriptional fusion with a 254-bp fragment containing the Pc variant and the P2 promoter region (see Materials and Methods). We found, in agreement with the results of a previous study [11] and those of another study published during the course of this work [13], that PcS was about 25-fold stronger than PcW and 4.5-fold stronger than PcH1, while PcH2 lay between PcH1 and PcS, being 3.8-fold stronger than PcH1. PcPUO and PcIn42 were of similar strength to PcW, and PcIn116 was very weak (Figure 2A). The PcSS variant, previously described as being stronger than PcS [14], was about 12-fold less efficient in our experimental conditions (Figure 2A). This latter result was not wholly unexpected, as PcSS contains a down-promoter mutation in the −35 hexamer relative to PcS (Table 1; [21]).

We found that the presence of the TGN-10 motif increased PcW efficiency 15-fold, approaching that of PcH2, whereas it had no significant effect on PcS or PcH2 activity (Figure 2B), probably because these promoters are already maximally efficient. On the other hand, the C to A mutation in PcW_TAN-10_ severely reduced PcW activity (as already observed for the activity of an *Escherichia coli* promoter [19]), and the C to T mutation in PcH1_TTN-10_ slightly increased PcH1 efficiency (1.7-fold; data not shown).

To evaluate the contribution of P2 to gene cassette expression, we first created transcriptional *lacZ* fusion with sequences containing a combination of an inactive PcS (hereafter named PcS*, see Materials and Methods) and the P2 variants, in order to assess their specific strength. We found that P2 was active and 7-fold stronger than PcW (Figure 2C), in keeping with previous studies [11]. P2m1 and P2m2 appeared to be inactive (data not shown) and their influence on gene cassette expression was not investigated further. When the weakest Pc variants (PcW and PcH1) were associated with P2, β-galactosidase activity was increased but was equivalent to that of P2, indicating that, in the PcW-P2 and PcH1-P2 combinations, PcW and PcH1 do not contribute significantly to the expression of gene cassettes, which is mainly driven by P2. By contrast, when P2 was associated with the strongest variants, PcS and PcW_TGN-10_, β-galactosidase activity decreased slightly (Figure 2C). A recent report described a small increase in the expression of a gene cassette when PcS was combined with P2 [13], but these authors used different methods to measure promoter strength, which may explain the discrepancy with our results.

### The nature of the Pc variant defines several IntI1 variants

In class 1 MRIs, Pc is located within the *intI1* coding sequence, and several of the substitutions generating the different Pc variants affect the IntI1 amino acid (aa) sequence. The aa changes involve aa 32 or 39 for the main variants and aa 31, 32, 38 and/or 39 for the rare variants (Table 3). Some Pc variants produce the same IntI1 variant, e.g. PcW/PcH1 and PcS/PcH2 (Table 3). Altogether, 10 IntI1 variants are generated from 13 Pc variants, three of which (IntI1_R32_H39_, IntI1_R32_N39_ and IntI1_P32_H39_) represent almost 96% of the IntI1 variants (Table 3).

**Table 3:** Correlation between the Pc variant configuration and amino acid changes in the integrase sequence.

IntI1 name	Pc variant^a^	aa 31^b^	aa 32^b^	aa 38^b^	aa 39^b^	Frequency (%)^c^
IntI1_R32_H39_	PcW, PcH1	L	R	V	H	51.4
IntI1_R32_N39_	PcS, PcH2	L	R	V	**N**	26.8
IntI1_P32_H39_	PcW_TGN-10_	L	**P**	V	H	17.4
IntI1_P32_N39_	PcS_TGN-10_, PcH2_TGN-10_	L	**P**	V	**N**	1.9
IntI1_L32_H39_	PcW_TAN-10_	L	**L**	V	H	0.3
IntI1_Q32_H39_	PcH1_TTN-10_	L	**Q**	V	H	0.6
IntI1_R32_I38_N39_	PcSS	L	R	**I**	**N**	0.3
IntI1_R32_A38_N39_	PcIn42	L	R	**A**	**N**	0.3
IntI1_R32_D38_N39_	PcPUO	L	R	**D**	**N**	0.6
IntI1_S31_R32_N39_	PcIn116	**S**	R	V	**N**	0.3

**a** Pc variant present in the *intI1* coding sequence.

**b** Nature of the amino acids corresponding to aa 31, 32, 38 and 39 of IntI1. Amino acids differing from those corresponding to the translation of the *intI1* sequence containing PcW are shown in bold.

**c** Frequency among the 321 integrons analyzed.

### The different IntI1 variants display a wide range of excision activities but similar integration activities

**Figure 3:**
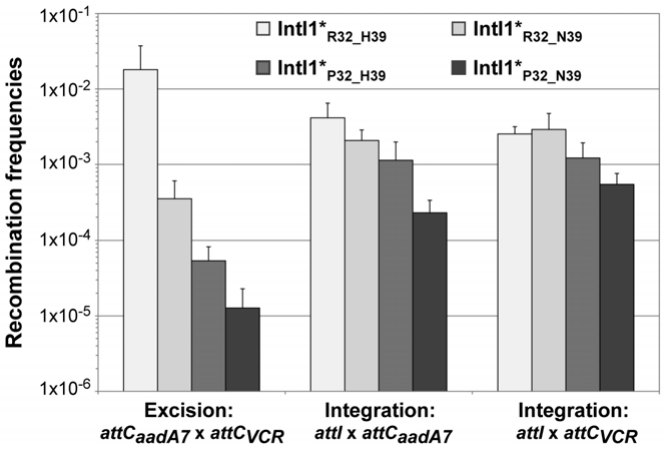
Recombination activities of the main IntI1 variants. IntI1 excision recombination activity was estimated by determining the frequency of emergence of the tobramycin resistance phenotype as a result of recombination between the *attC* sites of the *attC_aadA7_-catT4-attC_VCR_-aac(6′)-Ib* array, leading to the deletion of the synthetic cassette *catT4-attC_VCR_* and expression of the tobramycin resistance gene *aac(6′)-Ib*. IntI1 integration recombination activity was estimated by determining the frequency of emergence of cointegrate of two plasmids, one carrying an *attI* site and the other an *attC* site. Error bars indicate the standard error of the mean for at least seven independent assays.

In order to estimate the impact of the aa differences on IntI1 activity, we first cloned the *intI1* gene of the three main IntI1 variants, IntI1_R32_H39_, IntI1_R32_N39_ and IntI1_P32_H39_, under the control of the arabinose-inducible promoter P*araB* (see Materials and Methods). However, we anticipated that the two convergent promoters, namely Pc (contained in the *intI* sequence) and P*araB*, might interfere with each other. Thus, to estimate IntI1 protein recombination activity independently of potential promoter interference, we introduced mutations that inactivated the Pc promoters without affecting the IntI1 aa sequence (see Materials and Methods). The resulting integrases were named IntI1*_R32_H39_, IntI1*_R32_N39_ and IntI1*_P32_H39_ (Table 3). We then estimated the excision activity of these integrases by measuring their capacity to catalyze recombination between two *attC* sites located on a synthetic array of two cassettes, *attC_aadA7_-cat(T4)-attC_VCR_-aac(6′)-Ib*, and resulting in the deletion of the synthetic cassette, *cat(T4)-attC_VCR_*, and the expression of tobramycin resistance mediated by the gene *aac(6′)-Ib* (see Materials and Methods; [22]). As shown in Figure 3, the three integrases exhibited very different excision activities (1.8×10^−2^ to 1.3×10^−5^), IntI1*_P32_H39_ and IntI1*_R32_N39_ being respectively 336- and 51-fold less efficient than IntI1*_R32_H39_. Thus, replacing R32 by P32, or H39 by N39 drastically reduces the capacity of the integrase to promote recombination between the *attC_aadA7_* and *attC_VCR_* sites. The strongest effect was observed when a proline was present at position 32. P32 is also found in the integrase IntI1_P32_N39_, a much less frequent variant of IntI1 (Table 3). We therefore created this latter IntI1 variant and measured its excision activity. IntI1*_P32_N39_ was 27-fold less active than IntI1*_R32_N39_, showing the same negative effect of P32 on excision activity (Figure 3).

Class 1 integrase is also able to catalyze the integration of gene cassettes by promoting recombination between *attI* and *attC* sites [5]. We therefore tested the ability of the different IntI1 variants to catalyze recombination between *attI* and the two *attC* sites used for the excision activity assay (*attC_aadA7_* and *attC_VCR_*), in an assay based on suicide conjugative transfer previously developed [6] and since extensively used [23]–[25] (see Materials and Methods). Surprisingly, the range of integration activity of the four IntI variants tested in this study was rather narrow (4.5×10^−3^ to 2.3×10^−4^) compared to their excision activity, independently of the nature of the *attC* site (Figure 3). IntI1*_R32_H39_ and IntI1*_R32_N39_ exhibited similar integration activities in the two reactions performed, and the R32P substitution appeared to be detrimental for the activity of both integrases, but far less than for their excision activity. This effect seemed a bit stronger with IntI1*_P32_N39_ than with IntI1*_P32_H39_ (integration frequency was reduced by roughly 8-fold compared to 3-fold, respectively; Figure 3).

To show that the observed differences in excision and integration activities of the four integrases tested were not due to variations in the amounts of integrase but indeed to the nature of the aa at positions 32 and 39, we performed SDS-Page western blot analysis. We found that IntI1*_R32_H39_, IntI1*_R32_N39_ and IntI1*_P32_N39_ were equally produced and that IntI1*_P32_H39_ was slightly more strongly expressed in our experimental conditions (Figure S1 and Text S1). However, the latter had one of the weakest recombination activities (Figure 3). Therefore, the observed differences in excision activity among the IntI1 variants were due not to differences in protein abundance but to differences in protein activity and/or folding.

## Discussion

In this study we found marked polymorphism of the gene cassette promoter Pc (13 variants), corresponding to ten variants of the class 1 integrase IntI1. The 13 Pc variants were defined on the basis of the −35 and −10 hexamers and the sequence upstream of the −10 box. Indeed almost 20% of the 321 integrons analyzed here harbored a TGN-10 motif that characterized an extended −10 promoter. This feature was mainly associated with the weak PcW variant (41.8% of PcW-containing integrons) and increased the efficiency of this promoter by a factor of 15. In view of its frequency and its strength difference relative to PcW, we propose that this promoter, designated PcW_TGN-10_, be considered as a Pc variant distinct from PcW. Furthermore, 9% of the 321 integrons contained the P2 promoter, which was almost exclusively associated with the PcW variant (17.2% of PcW-containing integrons, Table 2). As in previous studies, we found that transcriptional activity was mainly driven by P2 in the PcW-P2 combination [9],[11]. We also observed the same effect with PcH1.

Altogether, there are no fewer than 20 distinct gene cassette promoter configurations for class 1 integrons, but their frequencies are very different. Five main combinations emerged from the dataset, defining five levels of promoter strength. The distribution and strength of the gene cassette promoters were as follows: PcW-P2<PcW≈PcW_TGN-10_<PcS≈PcH1 (distribution, Table 2) and PcW<PcH1<PcW-P2<PcW_TGN-10_<PcS (respectively 4.5-, 7-, 15- and 25-fold more active than PcW; Figure 1 and Figure 2).

The multiplicity of gene-cassette promoters displaying different strengths indicates that a given antibiotic resistance gene cassette will be differently expressed depending on which Pc variant is present in the integron. For example, we used an *E. coli* strain containing a class 1 integron with PcW, PcS or PcW_TGN-10_, and with *aac(6′)-Ib* as the first cassette. The tobramycin MIC was 8-fold higher when the cassette was expressed from PcS or PcW_TGN-10_ than from PcW (data not shown). Our findings indicate that, in class 1 integrons, gene cassette expression is mainly controlled by the strongest Pc variants (PcS, PcH2, PcW_TGN-10_ and PcW-P2, in 55% of cases).

Another important and previously unnoticed feature of class 1 integrons is the variability of the IntI1 primary sequence linked to the diversity of Pc variants. Among the 10 IntI1 variants identified, three (IntI1_R32_H39_, IntI1_R32_N39_ and IntI1_P32_H39_) accounted for almost 96% of class 1 integrases (Table 3). We found that these three main IntI1s displayed similar integration efficiencies, independently of the *attC* sites tested, whereas they had extremely different excision activities, depending on the nature of the amino acid at position 32 and/or 39. The R32P and H39N substitutions each drastically reduced the capacity of the integrase to promote recombination between the *attC_aadA7_* and *attC_VCR_* sites (by 336- and 51-fold, respectively). In the integrase of the *Vibrio cholerae* chromosomal integron VchIntIA, the aa found at the position equivalent to residue 32 is basic, while the aa at position equivalent to residue 39 is a histidine (K21 and H28, respectively [24]), showing that, among IntI1 variants, IntI1_R32_H39_ is its closest relative. The crystal structure of VchIntIA bound to an *attC* substrate showed that these amino acids are located within an α-helix involved in *attC* binding [26]. This α-helix is conserved in the predicted structure of IntI1 and presumably plays the same role in recombination [24]. Thus, mutations of aa 32 and 39 in IntI1 might perturb the binding and thus undermine the recombination efficiency of *attC*×*attC*. The positively charged aa R32 may also play a role in the interaction with the *attC* site in the *attI*×*attC* recombination reaction. Indeed, a R32P substitution in both IntI1*_R32_H39_ and IntI1*_R32_N39_ reduced the integration frequency, but to a lesser extent than in an excision reaction (Table 3 and Figure 3). In contrast, aa H39 does not seem to be involved in the integration reaction. The *attI*×*attC* and *attC*×*attC* recombination reactions may thus involve different regions of the integrase. Indeed, Demarre and collaborators isolated two IntI1_R32_H39_ mutants, IntI1_P109L_ and IntI1_D161G_, that showed much higher integration efficiencies [24].

Interestingly, we found a correlation between Pc strength and integrase excision activity: the weaker the Pc variant, the more active the IntI1. Among the four integrases tested, IntI1_R32_H39_, which was the most prevalent IntI1 in our dataset (Table 3), had the most efficient excision activity and also displayed higher excision than integration activity. Integrons with this integrase contain either the PcW variant, leading to a weak expression of the gene cassette array, or the PcH1 variant, associated with slightly higher expression (4.5-fold). PcW-containing integrons could compensate for a low level of antibiotic resistance expression by the high excision efficiency of IntI1_R32_H39_, which confers a marked capacity for cassette rearrangement, in order to place the required gene cassette closer to Pc. In a recent study, Gillings *et al* suggested that chromosomal class 1 integrons from environmental β-proteobacteria might be ancestors of current clinical class 1 integrons [27]. The integrons they described all encoded IntI1_R32_H39_ and contained the PcW variant. We suspect that, under antibiotic selective pressure, these “ancestor” integrons may have evolved to enhance gene cassette expression, without modifying the potential for cassette reorganization, either through a single mutation (conversion of PcW to PcH1) or by the creation of a second promoter, P2, that is seven times more active. The high frequency of PcH1 (27.3%) likely reflects its successful selection. P2 probably arises less frequently, as it requires the insertion of three G. We have recently shown that the expression of IntI1 is regulated *via* the SOS response, a LexA binding site overlapping its promoter [22]. Interestingly, when P2 is created, the insertion of three G disrupts the LexA binding site, probably leading to constitutive expression of IntI1.

In a context of stronger antibiotic selective pressure, the need to express gene cassettes more efficiently could have led to the selection of more efficient Pc sequences (such as PcS and PcW_TGN-10_) at the expense of IntI1 excision activity, resulting in the stabilization of successful cassette arrays. This hypothesis is consistent with the observation that integrons bearing IntI1_R32_N39_ or IntI1_P32_H39_ tend to harbor larger gene cassette arrays than those bearing IntI1_R32_H39_ (Figure S2).

The tight relationship between the aptitude of class 1 integrons to recombine and to express gene cassettes may be one key to understanding short-term integrase evolution. Different antibiotic selective pressures might select different evolutionary compromises. Thus, integron-driven drug resistance is more complex than previously thought.

## Materials and Methods

### Genbank class 1 integron sequence analysis

Compilation of the class 1 promoter sequences was performed in the entire Genbank nucleotide collection (nr/nt) using the alignment search tool BLASTn (http://www.ncbi.nlm.nih.gov/BLAST) and the sequences of the *intI1* and/or *attI1* from the In40 integron as reference [28] (GenBank accession number AF034958). This data extraction was performed on 2009-02-01. Three other published but non-deposited sequences [14],[29],[30] were added to the 1351 sequences collected above. Of these 1351 sequences, only 434 contained both the Pc and P2 promoter sequences. Among the latter 434 sequences, we identified the integrons that displayed both identical gene cassette arrays and identical Pc/P2 sequences, independently of their bacterial origin. This analysis led to the isolation of 321 unique class 1 integron sequences that were further studied (Table S1).

### Bacteria and growth conditions

The bacterial strains and plasmids are listed in Table 4. Cells were grown at 37°C in brain-heart infusion broth (BHI) or Luria Bertani broth (LB) supplemented when necessary with kanamycin (Km, 25 µg/ml), ampicillin (Amp, 100 µg/ml), tobramycin (Tobra, 10 µg/ml), chloramphenicol (Cm 25 µg/ml), DAP (0.3 mM), glucose (1%), arabinose (0.2%).

**Table 4:** Bacterial strains and plasmids used in this study.

Strain/ plasmid	Name	Genotype or description	Source or reference
***E. coli*** ** strains**	MC1061	*hsdR2 hsdM^+^ hsdS^+^ araD139* Δ*(ara-leu)7697*Δ*(lac)X74 galE15galK16 rpsL (Str^R^) mcrA mcrB1*	Laboratory collection
	MG1656	MG1655Δ*lacMlu*I	[33]
	β2163	(F−) RP4-2-Tc::Mu Β*dapA*::(erm, pir) (Km^R^)	[32]
**Plasmids**	pAT674	6.5-kb BamHI fragment from In40 class 1 integron cloned into pBGS18; Carries a strong Pc variant.	[28]
	pRMH821	R388 derivative in which the strong Pc variant was replaced by a weak variant.	[9]
	pSU38*lacZ*α*_lacZ*	pSU38-derived, lacking the *lacZ*α gene.	[5]
	pSU18Δ	pSU18 with the *lacZ* promoter deleted.	[5]
	p6851	pSU38p*lac-attC_aadA7_-catT4-attC_VCR_-aac(6′)-Ib*	[22]
	pSU38Δtot*lacZ*	Vector carrying the *lacZ* coding sequence with no translation initiation region or promoter.	This study
	pPcS	int4b×ΔORF11 PCR product from pAT674 cloned into pSU38Δtot*lacZ*	This study
	pPcW	int4b×ΔORF11 PCR product from pRMH821 cloned into pSU38Δtot*lacZ*.	This study
	pPcH1	PcS mutated with primers 7 and 8 to create PcH1.	This study
	pPcH2	PcS mutated with primers 9 and 10 to create PcH2.	This study
	pPcPUO	PcS mutated with primers 5 and 6 to create PcPUO.	This study
	pPcSS	PcS mutated with primers 15 and 16 to create PcSS.	This study
	pPcIn42	PcS mutated with primers 11 and 12 to create PcIn42.	This study
	pPcIn116	PcS mutated with primers 13 and 14 to create PcIn116.	This study
	pPcS*	PcS mutated with primers 3 and 4 to inactivate PcS.	This study
	pPcS-TGN	PcS mutated with primers 19 and 20; C to G mutation 2 bp upstream of the −10 hexamer to create a TGN-10 extended motif in PcS.	This study
	pPcW-TGN	PcW mutated with primers 21 and 22; C to G mutation 2 bp upstream of the −10 hexamer to create a TGN-10 extended motif in PcW.	This study
	pPcH2-TGN	PcH2 mutated with primers 21 and 22; C to G mutation 2 bp upstream of the −10 hexamer to create a ‘TGN-10’ extended motif in PcH2.	This study
	pPcS-P2	Creation of a P2 promoter in pPcS using primers 25 and 26.	This study
	pPcW-P2	Creation of a P2 promoter in pPcW using primers 25 and 26.	This study
	pPcH1-P2	Creation of a P2 promoter in pPcH1 using primers 25 and 26.	This study
	pPcW_TGN-10_-P2	Creation of a P2 promoter in pPcW-TGN using primers 25 and 26.	This study
	pPcS*-P2	Inactivation of the PcS promoter in pPcS-P2 using primers 3 and 4.	This study
	pSU38-*attI*	pSU38Δ::*attI*	[5]
	pVCR-B	pSW23T::VCR_2/1_B; contains the *V. cholerae* VCR site; the bottom strand will be transferred by conjugation.	[6]
	pAttC-B	pSW23T_ISS_::*aadA7*-B; contains the *aadA7 attC* site; the bottom strand will be transferred by conjugation.	[6]
	p734	*intI1* containing the PcW variant cloned into pBAD18.	[24]
	pBad-intI1*_R32_H39_	encodes IntI1*_R32_H39_ (IntI1_R32_H39_ in which PcW is inactivated).	This study
	pBad-intI1*_R32_N39_	encodes IntI1*_R32_N39_ (intI1*_R32_H39_ mutated with primers 7 and 8, converts IntI1*_R32_H39_ to IntI1*_R32_N39_ (inactive PcS))	This study
	pBad-intI1*_P32_H39_	encodes IntI1*_P32_H39_ (intI1*_R32_H39_ mutated with primers 33 and 34, converts IntI1*_R32_H39_ to IntI1*_P32_H39_ (inactive PcW_TGN-10_)).	This study
	pBad-intI1*_P32_N39_	encodes IntI1*_P32_N39_ (intI1*_R32_N39_ mutated with primers 33 and 34, converts IntI1*_R32_N39_ to IntI1*_P32_N39_ (inactive PcS_TGN-10_)).	This study

### Assembly PCR

Mutations of the Pc and P2 promoter sequences were generated by assembly PCR with overlapping primers that contained the desired mutation and two external primers, int4b and ΔORF11 (Table 5). The two primary PCR products were then used in an equimolar ratio as templates for a second PCR step with the two external primers.

**Table 5:** Primers used in this study.

Primer number	Primer name	Sequence (5′-3′)^a^
1	Int4b	CCGGAATTCACACCGTGGAAACGGATGAAG
2	ΔORF11	CGCGGATCCATCGTTGCTGCTCCATAACA
3	MutL10	AGCCTGTTCGGTTCGCAGTGAGTAATGCAAGTAGCGTATGC
4	MutR10	CGCTACTTGCATTACTCACTGCGAACCGAACAGGCTTATGT
5	MutPcPUOL	AGGCACGAACCCAGTCGACATAAGCCTGTTCGG
6	MutPcPuoR	GAACAGGCTTATGTCGACTGGGTTCGTGCCTTC
7	MutPcH1L	AGGCACGAACCCAGTGGACATAAGCCTGTTCGG
8	MutPcH1R	GAACAGGCTTATGTCCACTGGGTTCGTGCCTTC
9	MutPcH2L	AGCCTGTTCGGTTCGTAAGCTGTAATGCAAGTAGCGTATGC
10	MutPcH2R	CGCTACTTGCATTACAGCTTACGAACCGAACAGGCTTATGT
11	MutPcIn42L	AGGCACGAACCCAGTTGGCATAAGCCTGTTCGG
12	MutPcIn42R	GAACAGGCTTATGCCAACTGGGTTCGTGCCTTC
13	MutPcIn116L	AGCCTGTTCGGTTCGTGAACTGTAATGCAAGTAGCGTATGC
14	MutPcIn116R	CGCTACTTGCATTACAGTTCACGAACCGAACAGGCTTATGT
15	MutPcSSL	AGGCACGAACCCAGTTGATATAAGCCTGTTCGG
16	MutPcSSR	GAACAGGCTTATATCAACTGGGTTCGTGCCTTC
17	MutPcW-14AL	TAAGCCTGTTCGGTTAGTAAGCTGTAATGCAAGTAGCGTAT
18	MutPcW-14AR	CGCTACTTGCATTACAGCTTACTAACCGAACAGGCTTATGT
19	MutPcS-14GL	TAAGCCTGTTCGGTTGGTAAACTGTAATGCAAGTAGCGTAT
20	MutPcS-14GR	CGCTACTTGCATTACAGTTTACCAACCGAACAGGCTTATGT
21	MutPcW-14GL	TAAGCCTGTTCGGTTGGTAAGCTGTAATGCAAGTAGCGTAT
22	MutPcW-14GR	CGCTACTTGCATTACAGCTTACCAACCGAACAGGCTTATGT
23	MutPcS-14TL	TAAGCCTGTTCGGTTTGTAAACTGTAATGCAAGTAGCGTAT
24	MutPcS-14TR	CGCTACTTGCATTACAGTTTACAAACCGAACAGGCTTATGT
25	P2MutL	ATGACTGTTTTTTTGGGGTACAGTCTATGCCTCGGGCATCCAAG
26	P2MutR	ATGCCCGAGGCATAGACTGTACCCCAAAAAAACAGTCATAACAA
27	P2Mut1L	CTGTTTTTTTTGGGGGACAGTCTATGCCTCGGGCAT
28	P2Mut1R	CCCGAGGCATAGACTGTCCCCCAAAAAAAACAGTCA
29	P2Mut2L	CTGTTTTTTTGGGGTACACACTATGCCTCGGGCATCCAAG
30	P2Mut2R	CCCGAGGCATAGTGTGTACCCCAAAAAAACAGTCATAAC
31	IntI1-PvuII	CGACAGCTGCTCGCGCAGGCTGGG
32	IntI1-EcoRI	CCGGAATTCGAGCTCTAACAAAGGAGCAAGCCATGAAAACCGCCACTGCG
33	MutL10-TGN	AGCCTGTTCGGTTGGCAGTGAGTAATGCAAGTAGCGTATGC
34	MutR10-TGN	CGCTACTTGCATTACTCACTGCCAACCGAACAGGCTTATGT
35	Rev2	AGCGGATAACAATTTCACACAGGA
36	Cm-frt-Verif5	TTATACGCAAGGCGACAAGGT

**a** Pc and P2 hexamers are underlined and mutated bases are in bold.

### Plasmid construction

#### Reporter vector pSU38*ΔtotlacZ*

The SacII-EcoRI region of pSU38*lacZ*α*_lacZ*, which contains the promoter, the ribosome binding site (RBS) and the first codons of the *lacZ* gene, was replaced by the 255-bp SacII-EcoRI fragment of pSU18Δ [5] that contains the same first codons of *lacZ* but no transcriptional or translational signals.

#### *lacZ* transcriptional fusions

The PcS-*lacZ* and PcW-*lacZ* transcriptional fusions were constructed by cloning, into the EcoRI-BamHI sites of pSU38Δtot*lacZ*, a 264-bp fragment containing both the Pc and P2 sequences, amplified from pAT674 and pRMH821 respectively (Table 4). All the −*lacZ* fusions with the other Pc variant configurations or combinations were obtained by assembly PCR with specific primers, as described above. The pSU38Δtot*lacZ* fusion plasmids are listed in Table 4. All cloned fragments were verified by sequencing. Oligonucleotides were purchased from Sigma-Genosys and are listed in Table 5.

#### IntI1* expression vectors

The p734 plasmid carries the IntI1_R32_H39_ integrase under the control of the arabinose-inducible P*araB* promoter [5]. The Pc −10 box, which lies within *intI1* at positions corresponding to aa S30 and L31, was inactivated without modifying the IntI1 aa sequence, i.e. the codon mutation AGC to TCA conserved S30 and codon mutation TTA to CTG conserved L31. The Pc −10 box was inactivated by PCR assembly using p734 as template, internal primers 3 and 4, and external primers 31 and 32. The resulting plasmid, pBad-intI1*_R32_H39_, was used to create pBad-intI1*_R32_N39_ and pBad-intI1*_P32_H39_ by PCR assembly with the internal primers indicated in Table 4. Likewise, pBad-intI1*_R32_N39_ was used to create pBad-intI1*_P32_N39_. Cloned fragments were verified by sequencing.

### β-galactosidase assay

Each transcriptional fusion plasmid was transformed into *E. coli* strain MC1061 to measure β-galactosidase enzyme activity. Assays were performed with 0.5-ml aliquots of exponential-phase cultures (OD_600_ = 0.6–0.8) as described by Miller [31] except that the incubation temperature was 37°C. Experiments were done at least 5 times for each strain.

### Integrase excision activity assay

A synthetic array of two cassettes *attC_aadA7_-cat(T4)-attC_VCR_-aac(6′)-Ib* preceded by the *lac* promoter is carried on plasmid p6851. This construction confers chloramphenicol resistance from the *cat* gene encoding chloramphenicol acetyltransferase from *Tn*9, here followed by a phageT4 rho-independent terminator, to prevent transcriptional read-through. The excision assay is based on the capacity of the integrase to catalyze recombination between the *attC* sites, resulting in the deletion of the synthetic cassette *cat(T4)-attC_VCR_* and expression of the tobramycin resistance gene *aac(6′)-Ib* from the *lac* promoter [22]. IntI1 proteins were expressed from the pBad-intI1* plasmids. A stationary-phase liquid culture of *E. coli* strain MG1656, carrying both p6851 and one of the pBad-intI1*, grown over-day in LB broth supplemented with antibiotics and glucose, was diluted 100-fold in LB broth supplemented with antibiotics plus either glucose or arabinose and was grown overnight. Recombinants were selected on LB-Tobra plates. Excision frequency was measured by determining the ratio of Tobra^R^ to Km^R^ colonies.

### Integrase integration activity assay

The assay was based on the method described in [6] and since extensively used [23]–[25]. Conjugation is used to deliver the *attC* site carried onto a suicide vector from the R6K-based pSW family [32] into a recipient cell expressing the IntI1 integrase and carrying the *attI* site on a pSU38 plasmid derivative (all plasmids are listed in Table 4). Briefly, the RP4(IncPα) conjugation system uses the donor strain β2163 and the recipient MG1656, which does not carry the *pir* gene, and thus cannot sustain replication of pSW plasmids after conjugation. Recombination between *attI* and *attC* sites within the recipient cell leads to the formation of cointegrates between pSW and pSU38 plasmid. The number of recipient cells expressing the pSW marker (Cm^R^) directly reflects the frequency of cointegrate formation. IntI1 proteins were expressed from the pBad-intI1* plasmids. Conjugation experiments were performed as previously described [5]. Integration activity was calculated as the ratio of transconjugants expressing the pSW marker Cm^R^ to the total number of recipient Km^R^ clones. *attC-attI* cointegrate formation was checked by PCR with appropriate primers (primers 35 and 36; Table 5) on two randomly chosen clones per experiment. Background values were established by using recipient strains containing an empty pBad in place of the pBad-intI1*, and were 6×10^−7^ and 6×10^−8^ for the *attI*×*attC_VCR_* and *attI*×*attC_VCR_* assays, respectively. At least five experiments were performed for each recombination assay.

## Supporting Information

Figure S1 Expression level of the different IntI1 variants. The expression levels were estimated by western blot analysis from cell cultures. The IntI1 variants were expressed from the pBad-intI1* vectors induced by arabinose, the variants being indicated under the graph. T: purified IntI1_R32; H39_.
(1.07 MB EPS)

Figure S2 Distribution of the number of gene cassettes in arrays depending on the Pc variant. The number of gene cassettes was determined for the 321 integrons analyzed. The results were sorted according to the Pc variant controlling the cassette array transcription: PcW (n = 77), PcH1 (n = 88), PcW_TGN-10_ (n = 56), and PcS (n = 77).
(0.34 MB TIF)

Table S1 List and characteristics of the 321 analyzed integrons.
(0.30 MB DOC)

Text S1 Supporting materials and methods: integrase protein quantification.
(0.03 MB DOC)
