# Supplementary figures and images for: Inverse Correlation between Promoter Strength and Excision Activity in Class 1 Integrons
Source: PLoS Genet. 2010 Jan 8;6(1):e1000793. doi: 10.1371/journal.pgen.1000793 (PMC2791841; doi:10.1371/journal.pgen.1000793)

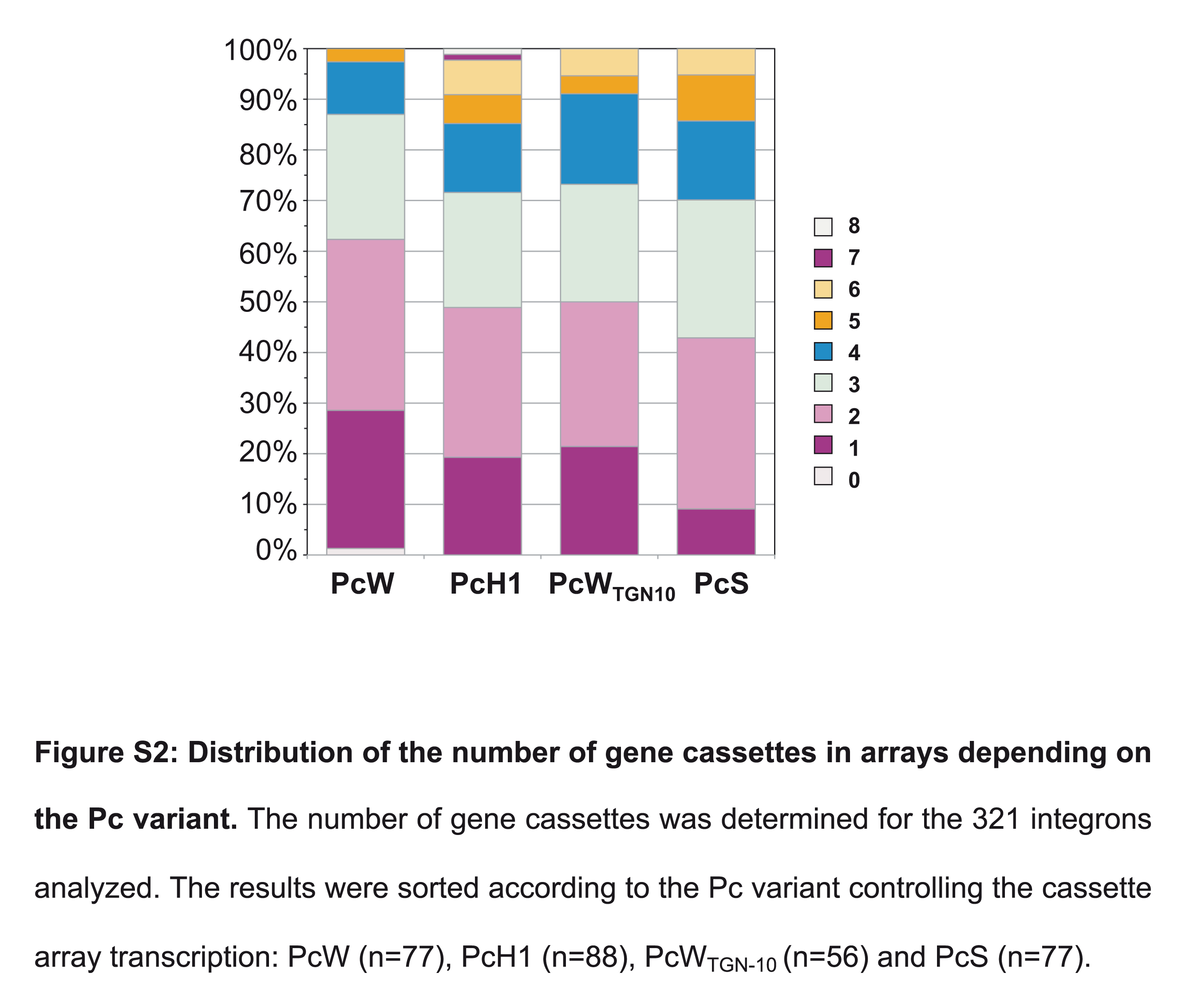

Supplement: Figure S2 — Distribution of the number of gene cassettes in arrays depending on the Pc variant. The number of gene cassettes was determined for the 321 integrons analyzed. The results were sorted according to the Pc variant controlling the cassette array transcription: PcW (n = 77), PcH1 (n = 88), PcWTGN-10 (n = 56), and PcS (n = 77). (0.34 MB TIF) [file pgen.1000793.s002.tif]
