# Supplementary material for: Inverse Correlation between Promoter Strength and Excision Activity in Class 1 Integrons
Source: PLoS Genet. 2010 Jan 8;6(1):e1000793. doi: 10.1371/journal.pgen.1000793 (PMC2791841; doi:10.1371/journal.pgen.1000793)
Supplement: Table S1 — List and characteristics of the 321 analyzed integrons. (0.30 MB DOC) [file pgen.1000793.s003.doc]

**Table S1: List and characteristics of the 321 analyzed integrons**

| **Pc variant** | **P2 form** | **Number of Gene Cassettes** | **Accession Number** |
| --- | --- | --- | --- |
| PcW |  | 0 | U49101, M73819 |
| PcW |  | 1 | L06157 |
| PcW |  | 1 | D43625 |
| PcW |  | 1 | AJ420072 |
| PcW |  | 1 | FJ457611 |
| PcW |  | 1 | AY257539 |
| PcW |  | 1 | X12868 |
| PcW |  | 1 | X58425 |
| PcW |  | 1 | EU598463 |
| PcW |  | 1 | DQ352176 |
| PcW |  | 1 | AB281182 |
| PcW |  | 1 | BX248359 |
| PcW |  | 1 | X53635 |
| PcW |  | 1 | AF191564 |
| PcW |  | 1 | EU327990 |
| PcW |  | 2 | EU327991 |
| PcW |  | 2 | AJ877225 |
| PcW |  | 2 | EF614235 |
| PcW |  | 2 | DQ767903 |
| PcW |  | 2 | EU503121 |
| PcW |  | 2 | AF024602 |
| PcW |  | 2 | AJ289190 |
| PcW |  | 2 | AF313471 |
| PcW |  | 2 | CP000645 |
| PcW |  | 2 | AM261282 |
| PcW |  | 2 | Z50802 |
| PcW |  | 2 | AJ628353 |
| PcW |  | 2 | CP001232 |
| PcW |  | 2 | EF592570 |
| PcW |  | 2 | DQ372711 |
| PcW |  | 2 | AJ863570 |
| PcW |  | 2 | AJ564903 |
| PcW |  | 3 | EU886977 |
| PcW |  | 3 | EU675686 |
| PcW |  | 3 | DQ914960 |
| PcW |  | 3 | L36547 |
| PcW |  | 3 | EU886979 |
| PcW |  | 3 | AF313472 |
| PcW |  | 3 | AY214164 |
| PcW |  | 3 | EU487199 |
| PcW |  | 3 | Z21672 |
| PcW |  | 3 | DQ393569 |
| PcW |  | 3 | AJ698325 |
| PcW |  | 3 | EF027105 |
| PcW |  | 3 | DQ236170 |
| PcW |  | 3 | AY115475 |
| PcW |  | 3 | EF577408 |
| PcW |  | 3 | EU327989 |
| PcW |  | 4 | AJ971344 |
| PcW |  | 4 | DQ522237 |
| PcW |  | 4 | DQ831140 |
| PcW |  | 4 | AY536743 |
| PcW |  | 5 | EU327987 |
| PcW | P2 | 1 | X12870 |
| PcW | P2 | 1 | CP000603 |
| PcW | P2 | 1 | X64368 |
| PcW | P2 | 1 | EF660563 |
| PcW | P2 | 1 | EF190326 |
| PcW | P2 | 1 | AF329699 |
| PcW | P2 | 1 | U81136 |
| PcW | P2 | 2 | AJ878850 |
| PcW | P2 | 2 | AF231133 |
| PcW | P2 | 2 | AY136758 |
| PcW | P2 | 2 | AF315786 |
| PcW | P2 | 2 | EU052800 |
| PcW | P2 | 2 | AF174129 |
| PcW | P2 | 2 | AF326777 |
| PcW | P2 | 2 | AJ746361 |
| PcW | P2 | 2 | AY289608 |
| PcW | P2 | 3 | AY758206 |
| PcW | P2 | 3 | AM055748 |
| PcW | P2 | 3 | AF347074 |
| PcW | P2 | 4 | EF636461 |
| PcW | P2 | 4 | AJ584652 |
| PcW | P2 | 4 | AJ969235 |
| PcW | P2 | 5 | AF156486 |
| PcW | P2m2 | 4 | [1] |
| PcW_TGN-10_ |  | 1 | CT025832 |
| PcW_TGN-10_ |  | 1 | AF261825 |
| PcW_TGN-10_ |  | 1 | AF071555 |
| PcW_TGN-10_ |  | 1 | AF486817 |
| PcW_TGN-10_ |  | 1 | AF071555 |
| PcW_TGN-10_ |  | 1 | EU847537 |
| PcW_TGN-10_ |  | 1 | FJ183463 |
| PcW_TGN-10_ |  | 1 | AB263754 |
| PcW_TGN-10_ |  | 1 | EU847537 |
| PcW_TGN-10_ |  | 1 | AB207867 |
| PcW_TGN-10_ |  | 1 | AM886293 |
| PcW_TGN-10_ |  | 1 | EF427691 |
| PcW_TGN-10_ |  | 2 | AY963803 |
| PcW_TGN-10_ |  | 2 | AY522431 |
| PcW_TGN-10_ |  | 2 | AM295980 |
| PcW_TGN-10_ |  | 2 | EF408254 |
| PcW_TGN-10_ |  | 2 | AB207867 |
| PcW_TGN-10_ |  | 2 | AY069972 |
| PcW_TGN-10_ |  | 2 | X17477 |
| PcW_TGN-10_ |  | 2 | CT025832 |
| PcW_TGN-10_ |  | 2 | AY146989 |
| PcW_TGN-10_ |  | 2 | AB366440 |
| PcW_TGN-10_ |  | 2 | AB469046 |
| PcW_TGN-10_ |  | 2 | EF118171 |
| PcW_TGN-10_ |  | 2 | AF133699 |
| PcW_TGN-10_ |  | 2 | DQ023222 |
| PcW_TGN-10_ |  | 3 | DQ219465 |
| PcW_TGN-10_ |  | 3 | DQ315789 |
| PcW_TGN-10_ |  | 3 | DQ522234 |
| PcW_TGN-10_ |  | 3 | DQ322593 |
| PcW_TGN-10_ |  | 3 | AY522923 |
| PcW_TGN-10_ |  | 3 | AM040708 |
| PcW_TGN-10_ |  | 3 | DQ902344 |
| PcW_TGN-10_ |  | 3 | FJ384365 |
| PcW_TGN-10_ |  | 3 | AF322577 |
| PcW_TGN-10_ |  | 3 | AJ867812 |
| PcW_TGN-10_ |  | 3 | AJ511268 |
| PcW_TGN-10_ |  | 3 | AM392427 |
| PcW_TGN-10_ |  | 3 | DQ342344 |
| PcW_TGN-10_ |  | 4 | EU886980 |
| PcW_TGN-10_ |  | 4 | AY943084 |
| PcW_TGN-10_ |  | 4 | AJ871915 |
| PcW_TGN-10_ |  | 4 | EF051038 |
| PcW_TGN-10_ |  | 4 | AY920928 |
| PcW_TGN-10_ |  | 4 | AJ609296 |
| PcW_TGN-10_ |  | 4 | EF125010 |
| PcW_TGN-10_ |  | 4 | AF078527 |
| PcW_TGN-10_ |  | 4 | Y18050 |
| PcW_TGN-10_ |  | 4 | AY560837 |
| PcW_TGN-10_ |  | 5 | AJ971342 |
| PcW_TGN-10_ |  | 5 | AJ634050 |
| PcW_TGN-10_ |  | 6 | AY907717 |
| PcW_TGN-10_ |  | 6 | EF051037 |
| PcW_TGN-10_ |  | 6 | CT025832 |
| PcW_TGN-10_ | P2 | 2 | AJ009819/AJ310778 |
| PcW_TGN-10_ | P2m1 | 2 | DQ315788 |
| PcW_TAN-10_ |  | 2 | AB040994 |
| PcH1 |  | 1 | FJ594767 |
| PcH1 |  | 1 | AF439785 |
| PcH1 |  | 1 | DQ247972 |
| PcH1 |  | 1 | AJ517791 |
| PcH1 |  | 1 | AF458081 |
| PcH1 |  | 1 | DQ108615 |
| PcH1 |  | 1 | FJ223605 |
| PcH1 |  | 1 | AJ517790 |
| PcH1 |  | 1 | EU715254 |
| PcH1 |  | 1 | U31119 |
| PcH1 |  | 1 | FJ167861 |
| PcH1 |  | 1 | EU491959 |
| PcH1 |  | 1 | CP000744 |
| PcH1 |  | 1 | AJ586617 |
| PcH1 |  | 1 | AM778842 |
| PcH1 |  | 2 | AF355189 |
| PcH1 |  | 2 | AB366442 |
| PcH1 |  | 2 | AY033653 |
| PcH1 |  | 2 | AF043381 |
| PcH1 |  | 2 | AJ561197 |
| PcH1 |  | 2 | AJ639924 |
| PcH1 |  | 2 | AY509004 |
| PcH1 |  | 2 | Z83311 |
| PcH1 |  | 2 | FJ183470 |
| PcH1 |  | 2 | AY259085 |
| PcH1 |  | 2 | AY878718 |
| PcH1 |  | 2 | AM886293 |
| PcH1 |  | 2 | AM746675 |
| PcH1 |  | 2 | CP000971 |
| PcH1 |  | 2 | DQ125241 |
| PcH1 |  | 2 | AB113580 |
| PcH1 |  | 2 | AB116260 |
| PcH1 |  | 2 | AJ628135 |
| PcH1 |  | 2 | AJ550807 |
| PcH1 |  | 2 | EF184215 |
| PcH1 |  | 2 | EU591509 |
| PcH1 |  | 2 | AF315351 |
| PcH1 |  | 2 | EF577407 |
| PcH1 |  | 2 | DQ522235 |
| PcH1 |  | 3 | EU886981 |
| PcH1 |  | 3 | AY775051 |
| PcH1 |  | 3 | AY878717 |
| PcH1 |  | 3 | AJ812570 |
| PcH1 |  | 3 | AY740681 |
| PcH1 |  | 3 | FJ594765 |
| PcH1 |  | 3 | AY536742 |
| PcH1 |  | 3 | EU678897 |
| PcH1 |  | 3 | EF382672 |
| PcH1 |  | 3 | AY551331 |
| PcH1 |  | 3 | DQ647028 |
| PcH1 |  | 3 | AB104852 |
| PcH1 |  | 3 | AF294653 |
| PcH1 |  | 3 | AJ786649 |
| PcH1 |  | 3 | EF207719 |
| PcH1 |  | 3 | EF207717 |
| PcH1 |  | 3 | DQ522236 |
| PcH1 |  | 3 | AY030343 |
| PcH1 |  | 3 | AY866525 |
| PcH1 |  | 3 | AY460181 |
| PcH1 |  | 4 | EF415651 |
| PcH1 |  | 4 | AY686225 |
| PcH1 |  | 4 | EU588392 |
| PcH1 |  | 4 | AJ620678 |
| PcH1 |  | 4 | AB188812 |
| PcH1 |  | 4 | DQ089809 |
| PcH1 |  | 4 | DQ839391 |
| PcH1 |  | 4 | M95287 |
| PcH1 |  | 4 | AJ867811 |
| PcH1 |  | 4 | AJ969237 |
| PcH1 |  | 4 | AJ969236 |
| PcH1 |  | 4 | EF522838 |
| PcH1 |  | 5 | AY260546 |
| PcH1 |  | 5 | AY444814 |
| PcH1 |  | 5 | EU219534 |
| PcH1 |  | 5 | AJ223604 |
| PcH1 |  | 5 | AY507153 |
| PcH1 |  | 6 | CP001125 |
| PcH1 |  | 6 | FJ160769 |
| PcH1 |  | 6 | FJ196385 |
| PcH1 |  | 6 | EU834941 |
| PcH1 |  | 6 | AY816216 |
| PcH1 |  | 6 | DQ393784 |
| PcH1 |  | 7 | EF184216 |
| PcH1 |  | 8 | AF047479 |
| PcH1 | P2 | 2 | AM991977 |
| PcH1 | P2 | 1 | AY522431 |
| PcH1 | P2 | 1 | EU266532 |
| PcH1 | P2m1 | 2 | EF184217 |
| PcH1_TTN-10_ |  | 2 | U36276 |
| PcH1_TTN-10_ |  | 2 | AF302086 |
| PcS |  | 1 | AY123253 |
| PcS |  | 1 | X74412 |
| PcS |  | 1 | EU219534 |
| PcS |  | 1 | AJ870926 |
| PcS |  | 1 | AJ628423 |
| PcS |  | 1 | EF690696 |
| PcS |  | 1 | AY781413 |
| PcS |  | 2 | AY458224 |
| PcS |  | 2 | EU543272 |
| PcS |  | 2 | AY162283 |
| PcS |  | 2 | FJ374756 |
| PcS |  | 2 | L06822 (X68227) |
| PcS |  | 2 | AB469045 |
| PcS |  | 2 | EF370423 |
| PcS |  | 2 | DQ861641 |
| PcS |  | 2 | AY463797 |
| PcS |  | 2 | DQ517526 |
| PcS |  | 2 | CP000604 |
| PcS |  | 2 | AY232671 |
| PcS |  | 2 | AF458082 |
| PcS |  | 2 | EF592571 |
| PcS |  | 2 | BR000038 |
| PcS |  | 2 | X72585, U67194 |
| PcS |  | 2 | DQ274503 |
| PcS |  | 2 | DQ288156 |
| PcS |  | 2 | U59183 |
| PcS |  | 2 | EU091084 |
| PcS |  | 2 | AF458080 |
| PcS |  | 2 | CP000863 |
| PcS |  | 2 | EF690695 |
| PcS |  | 2 | AY648125 |
| PcS |  | 2 | AJ628983 |
| PcS |  | 2 | AJ515707 |
| PcS |  | 3 | AF263519 |
| PcS |  | 3 | EU014166 |
| PcS |  | 3 | FJ207466 |
| PcS |  | 3 | AY220520 |
| PcS |  | 3 | EF375699 |
| PcS |  | 3 | DQ153217 |
| PcS |  | 3 | DQ153218 |
| PcS |  | 3 | EF660562 |
| PcS |  | 3 | EU118148 |
| PcS |  | 3 | EU118149 |
| PcS |  | 3 | AY816215 |
| PcS |  | 3 | AM993098 |
| PcS |  | 3 | DQ310703 |
| PcS |  | 3 | AJ311891 |
| PcS |  | 3 | AY294333 |
| PcS |  | 3 | U37105 |
| PcS |  | 3 | AM183120 |
| PcS |  | 3 | EF093145 |
| PcS |  | 3 | AM988778 |
| PcS |  | 3 | EU014075 |
| PcS |  | 4 | AF550679 |
| PcS |  | 4 | U90945 |
| PcS |  | 4 | AF034958 |
| PcS |  | 4 | AY065966 |
| PcS |  | 4 | AF263520 |
| PcS |  | 4 | U12338/X15852 |
| PcS |  | 4 | AJ310480 |
| PcS |  | 4 | DQ112355 |
| PcS |  | 4 | AJ870988 |
| PcS |  | 4 | AF369871 |
| PcS |  | 4 | AF418284 |
| PcS |  | 4 | EF467306 |
| PcS |  | 5 | EF207718 |
| PcS |  | 5 | EU117233 |
| PcS |  | 5 | CP001182 |
| PcS |  | 5 | CT025832 |
| PcS |  | 5 | AJ009820 |
| PcS |  | 5 | DQ522239 |
| PcS |  | 5 | AJ704863 |
| PcS |  | 6 | AY029772 |
| PcS |  | 6 | AY509004 |
| PcS |  | 6 | EF206349 |
| PcS |  | 6 | EF138817 |
| PcS | P2 | 3 | DQ143913 |
| PcS_TGN-10_ |  | 3 | AJ640197 |
| PcH2 |  | 1 | AB061794 |
| PcH2 |  | 1 | S45954 |
| PcH2 |  | 2 | CP000650 |
| PcH2 |  | 2 | AM180753 |
| PcH2 |  | 3 | AJ744860 |
| PcH2 |  | 4 | DQ522233 |
| PcH2 |  | 4 | AJ704863 |
| PcH2 |  | 4 | AJ278514/5 |
| PcH2 |  | 5 | U13880 |
| PcH2_TGN-10_ |  | 1 | AM234698 |
| PcH2_TGN-10_ |  | 1 | DQ519091 |
| PcH2_TGN-10_ |  | 2 | AY038837 |
| PcH2_TGN-10_ |  | 4 | FJ594766 |
| PcH2_TGN-10_ |  | 4 | AF445082 |
| PcPUO |  | 4 | AF288045 |
| PcPUO |  | 1 | S68049 |
| PcSS |  | 1 | [2] |
| PcIn116 |  | 3 | AJ621187 |
| PcIn42 |  | 3 | AJ243491 |

1. Lindstedt BA, Heir E, Nygard I, Kapperud G (2003) Characterization of class I integrons in clinical strains of Salmonella enterica subsp. enterica serovars Typhimurium and Enteritidis from Norwegian hospitals. J Med Microbiol 52: 141-149.

2. Brizio A, Conceicao T, Pimentel M, Da Silva G, Duarte A (2006) High-level expression of IMP-5 carbapenemase owing to point mutation in the -35 promoter region of class 1 integron among Pseudomonas aeruginosa clinical isolates. Int J Antimicrob Agents 27: 27-31.
