# Supplementary material for: Inverse Correlation between Promoter Strength and Excision Activity in Class 1 Integrons
Source: PLoS Genet. 2010 Jan 8;6(1):e1000793. doi: 10.1371/journal.pgen.1000793 (PMC2791841; doi:10.1371/journal.pgen.1000793)
Supplement: Text S1 — Supporting materials and methods: integrase protein quantification. (0.03 MB DOC) [file pgen.1000793.s004.doc]

**Integrase protein quantification**

The amount of IntI1* variants in cells was estimated by Western blot. *E. coli strain* MG1656(pSU38-*attI)* cells expressing one of the IntI1* variants from pBad*-intI1** were grown over-day in LB broth supplemented with ampicillin, kanamycin and 1% glucose, at 37°C, then diluted 1:100 in fresh LB broth supplemented with ampicillin, kanamycin and 0.2% arabinose and grown at 37°C overnight. Cells were pelleted, resuspended in gel loading buffer, boiled, and subjected to 12% SDS-PAGE. Proteins were transferred onto PVDF membranes and the membranes were probed with polyclonal anti-IntI1 peptide antibodies. The immunoblots were revealed with the WesternDot^TM^625 detection system (Molecular Probes) and the signals were quantified with the quantity One® sofware (Biorad). Results are expressed as a percentage of IntI1*_R32; H39_.
